# Phytochemical Profiling and Biological Potential of *Prunus dulcis* Shell Extracts

**DOI:** 10.3390/plants12142733

**Published:** 2023-07-23

**Authors:** Talel Ben Khadher, Sameh Sassi-Aydi, Samir Aydi, Mohamed Mars, Jalloul Bouajila

**Affiliations:** 1 Laboratoire de Génie Chimique, Université de Toulouse, CNRS, INP, UPS, F-31062 Toulouse, France; talel.ben-khadher@univ-tlse3.fr; 2 Laboratory of Biodiversity and Valorization of Bioressources in Arid Zones, Faculty of Sciences, The University of Gabes, Zrig, Gabes 6072, Tunisia; sameh_sassi@yahoo.fr (S.S.-A.); samir.aydi@gmail.com (S.A.); marsmohamed@yahoo.fr (M.M.)

**Keywords:** *Prunus dulcis* shell extracts, chemical composition, biological activities, GC-MS, HPLC-DAD

## Abstract

*Prunus dulcis* is one of the most widely cultivated species in the world. Its fruit (almond) is rich in various nutritious and bioactive compounds that exert several beneficial effects. The aim of this study was to determine the chemical profile and evaluate the biological potential in vitro of almond shell extracts. The chemical analysis of shell extracts led to the identification of 15 compounds by HPLC-DAD, of which 11 were first detected in the almond plant. Twenty-six volatile compounds were identified by the GC-MS technique; among them, seven were firstly detected in the studied plant. For the biological activities, the extracts demonstrated moderate inhibition potential against the antioxidant, antidiabetic, and cytotoxic activities. The methanol extract at 50 µg/mL showed the highest antioxidant (45%) and antidiabetic activities (45% against alpha-glucosidase and 31% against alpha-amylase extracts), while the cyclohexane and dichloromethane at 50 µg/mL showed the highest cytotoxic activity towards Hela (32.2% with cyclohexane) and RAW 264-7 (45% with dichloromethane). Overall, these findings demonstrate the potential of almond shell extracts as a source of bioactive compounds that could be applied in the pharmaceutical and medical fields.

## 1. Introduction

Nowadays, fruits and their by-products are widely studied as a promising source of phytochemicals with various health benefits that have a high potential to be integrated into the pharmaceutical and cosmetic industries given their various pharmacological and pharmaceutical properties [1].

Nuts are a class of food rich with high lipids content. Its consumption provides us with a multitude of bioactive compounds that present several pharmacological properties. They are known for their distinguished flavor, and their consumption provides us with a wide array of bioactive molecules and nutrients [2,3].

*Prunus dulcis* (*P. dulcis*) is one of the most cultivated nuts species in the world. It belongs to the Rosaceae family. The almond fruit is a stone fruit comprising a kernel, skin, shell, and hull. It presents a high nutritional value given its richness with high quality fats (monounsaturated fatty acids (MUFA, 60%) such as palmitic, stearic, and palmitoleic acid; polyunsaturated fatty acids (PUFA, 30%) such as linoleic acid), protein, carbohydrates, fibers, and vitamins [2,3]. Numerous bioactive compounds have been reported in the fruit of *P. dulcis* such as phenolic acids (hydroxycinnamic acid, hydro benzoic acid sinapic acid, etc.), flavonoids (Catechin, epicatechin, cyanidin, etc.), lignans, stilbene, and terpenoids [1,3,4]. Various biological proprieties have been reported in the almond, such as antimicrobial, antioxidant, anti-inflammatory, anticancer, laxative, hepatoprotective, cardiometabolic protective, neotropic, sedative, hypnotic, anxiolytic, and neuroprotective effects [4].

Interestingly, the almond shell, which accounts for 33% of the total fruit mass and produces annually 0.8 to 1.7 million tons globally, has been traditionally utilized as animal feed. However, it remains less characterized among the other parts, and is traditionally used as animal food. It is rich in cellulose and lignin contents, suggesting its potential for further exploration [5].

This study aims to evaluate the biological potential of the obtained shell extracts and the antioxidant (DPPH), antidiabetic (alpha-glucosidase, alpha amylase), and cytotoxic activity (Hela, RAW 264.7). The phytochemical profile of the shell extracts was determined by HPLC-DAD and GC-MS techniques. To the best of our knowledge, the current study was the first to focus on evaluating the biological potential and the phytochemical composition of *Prunus dulcis* shell part. The obtained results showed that almond shell extracts demonstrated moderate inhibitor activity with the antioxidant, antidiabetic, and cytotoxic assays, and led to the identification of 15 compounds by HPLC-DAD. Among them, 11 molecules were firstly identified within the almond plant. Additionally, 26 more compounds were discerned utilizing the GC-MS technique, with seven of them also being identified for the first time in the almond plant.

## 2. Results

### 2.1. Extraction Yield

In this study, the maceration technique, using an increasing polarity organic solvent (cyclohexane, dichloromethane, ethyl acetate, methanol, and H_2_O), was used in the extraction of the phytochemical compounds. As presented in Table 1, the polar fractions (MeOH, H_2_O) presented higher yield values than the nonpolar fractions; the highest yield value was recorded with the H_2_O extract (2.22%). The EtOAc extract presented the lowest value (0.03%). These findings suggest that the shell part is relatively richer in polar compounds such as polyphenols and polysaccharides. Conversely, the nonpolar fraction, which included dichloromethane and cyclohexane extracts, presented low yield values ranging from 0.09% to 0.14%, respectively, suggesting the presence of nonpolar compounds, such as fatty acids, in the shell material. In this context, Sarwar et al. [6] studied the effect of different solvents in the yield extraction of two varieties of *P. dulcis* shell, using 80 or 100% MeOH and 80 or 100% EtOH. The results showed significant variation in the yield between the different solvents, with the highest recorded with the 80% MeOH (18.2% for the thick shell and 57.9% for the thin shell). In another study, Queirós et al. [1] obtained an extraction yield value similar to our findings (3.4% with the ethanol–water extract).

### 2.2. Determination of the Total Polyphenol Content (TPC)

**Figure 1:**
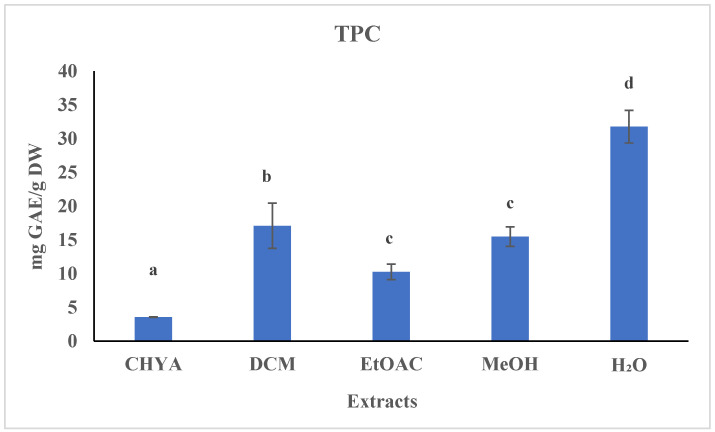
Total phenolic content (TPC) of *P. dulcis* shell extracts (Cyclohexane: CHYA; Dichloromethane: DCM; Ethyl acetate: EtOAc; Methanol: MeOH). Mean values ± SD (n = 3); Different letters on the histograms mean a significant difference (*p* < 0.05).

The Folin–Ciocalteu method was utilized to determine the TPC content in the shell fractions. As presented in the figure below (Figure 1), the polyphenol content of the obtained extracts is relatively low. Statistically, we noted a significant difference between the different extracts—the polar fractions have a higher TPC content than the apolar ones. The highest TPC content was recorded with the H_2_O extract (31.77 mg GAE/g of DW), while the lowest content was obtained with the CHYA extract (3.56 mg GAE/g of DW). In comparison to the literature, the values for phenolic content are higher than those found by Sarwar et al. [7]. Their study analyzed the methanolic and ethanolic shell extracts of two almond varieties. They reported values ranging from 1.4 to 2.3 mg GAE/g DW for the thick shell and from 2.3 to 7.21 mg GAE/g DW for the thin shell. In another study, Queirós et al. [6] obtained a higher TPC content (188.6 mg GAE/g DW) with the almond shell ethanol–water extract.

### 2.3. Determination of the Antioxidant Activity (DPPH) of Shell Extracts

**Figure 2:**
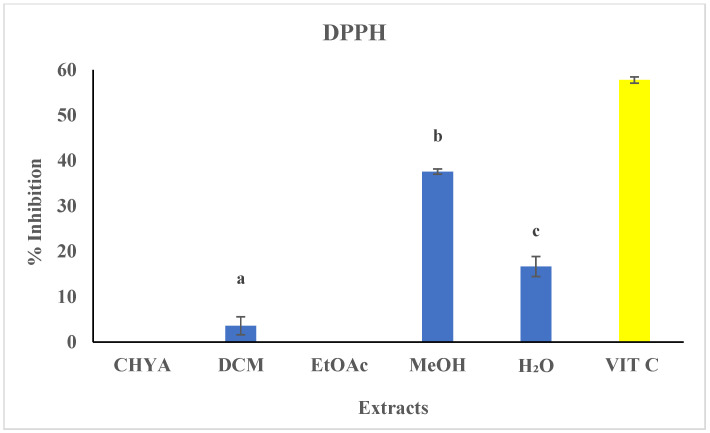
Antioxidant activity of *P. dulcis* shell extracts (Cyclohexane: CHYA; Dichloromethane: DCM; Ethyl acetate: EtOAc; Methanol: MeOH; ascorbic acid: VIT C: used as a reference at 4 µg/mL). Extracts were tested at 50 µg/mL. Mean values ± SD (n = 3); Different letters on the histograms mean a significant difference (*p* < 0.05).

The antioxidant potential of the almond shell extracts was determined using the DPPH assay. The shell extracts concentration was adjusted at 50 µg/mL, and ascorbic acid was used as reference at 4 µg/mL. The inhibitory assay result was illustrated in Figure 2. Statistically, there is a significant difference between the different extraction solvents. As shown in the figure below, only three extracts demonstrated antioxidant activity against the DPPH radical (DCM, MeOH, H_2_O). The highest inhibition values were observed in the polar extracts: 37.6% with the MeOH extract and 16.6% with the H_2_O extract. These findings suggest that polar molecules may be accountable for this observed effect. In fact, several polyphenolic compounds present in the almonds, such as catechin and epicatechin, are known for their antioxidant properties. These compounds could potentially synergize with other molecular classes such as fatty acids, vitamins such as A, B (thiamine B1, riboflavin B2, niacin B3, and pyridoxine B6), and E present in almonds [8], and polysaccharides (glycosides), which have also been found to possess antioxidant properties according to recent studies [8]. Compared to the literature, a study conducted by Queirós et al. [6], evaluating the antioxidant potential of almond shell extracts using the DPPH test, showed that the different extracts presented a moderate inhibition activity range of 12.1 to 18.22% for the thick shell and 15 to 57.9% for the thin shell. In another study, the DPPH assay was used to evaluate the antioxidant potential of almond shell extract. The results showed that the ethanol–water extract exerted a high inhibition activity, with IC_50_ equal to 7.9 µg/mL [1].

### 2.4. Chromatographic Analysis

#### 2.4.1. Identification of *P. dulcis* Shell Extracts Compounds by HPLC-DAD

**Figure 3:**
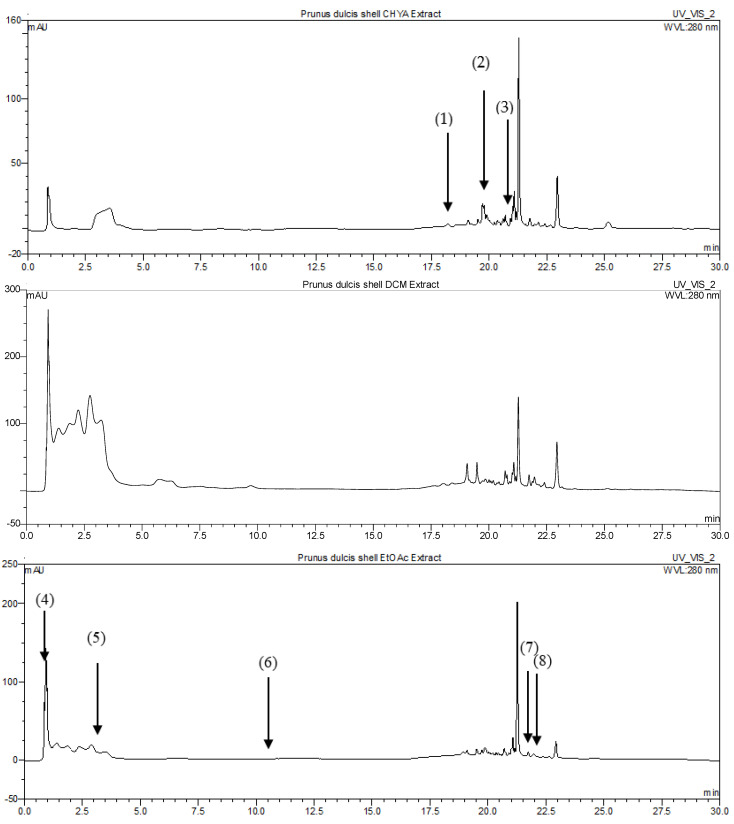

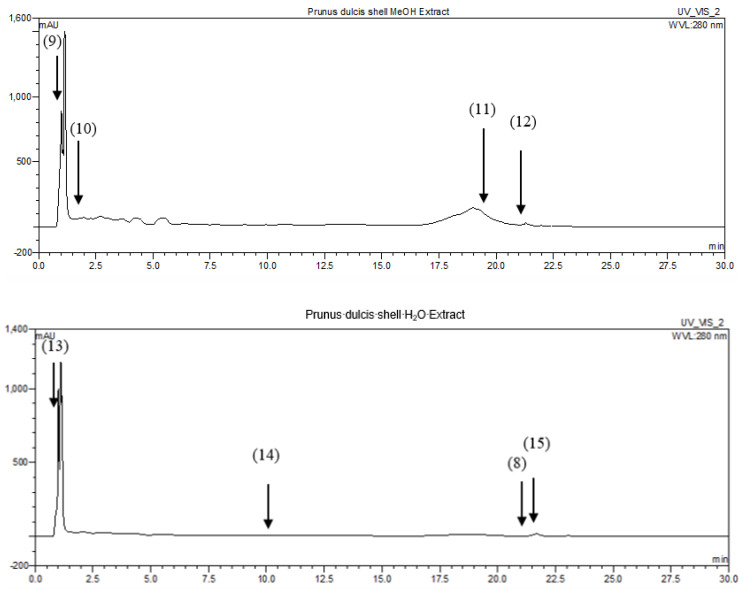
HPLC chromatograms of *P. dulcis* shell extracts. Peaks: (1): 5,7-dihydroxy-3′4′,5′-trimethoxyflavone; (2): 7-hydroxyflavone; (3): Isobutyl 4-hydroxybenzoate; (4): Rutin; (5): *trans*-3-hydroxycinnamic acid; (6): 7,8-dihydroxy-2,2-dimethylchromane-6-carboxylic acid; (7): 3,3′-dimethoxyflavone; (8): 3,6,3′-trimethoxyflavone; (9): Catechin; (10): Epicatechin; (11): 3-tert-butyl-4-hydroxybenzoic acid; (12): Shikonin; (13): Synephrine; (14): trans-cinnamic acid; (15): 5-hydroxy-3′-methoxyflavone.

The determination of compounds present within the almond shell extracts was performed using the HPLC-DAD technique. To determine the composition of the extracts, the retention time and maximum wavelength (λmax) of each peak were compared to those of standard compounds. These standards were injected under identical conditions to the shell extracts. After identification, each compound was quantified by referencing the corresponding calibration curve tailored to the specific standard. The analysis of the phytochemical composition of *P. dulcis* shell fractions led to the identification of 15 compounds. As presented in Figure 3 and Table 2, only four compounds were previously detected in the almond plant (catechin, epicatechin, rutin, and trans-cinnamic acid), while all the compounds were firstly identified in the shell part. The majority of the identified compounds were detected in the CHYA and the EtOAc extracts. The EtOAc extract was particularly rich in rutin, with a concentration of 13.06 mg per gram of extract. The methanol (MeOH) extract had a high content of epicatechin, at 4.42 mg/g extract, while the water (H_2_O) extract was notable for its high synephrine content, at 1.62 mg/g extract. According to the literature, few studies have been interested in the chemical composition of the almond shell, a study conducted by Moure et al. [9] identified vanillic, syringic, and p-coumaric acids in the *P. dulcis* shell part. In another study, Kahlaoui et al. [10] identified several polyphenolic compounds, such as quercetin-3-glucoside, p-coumaric acid, caffeic acid, and protocatechuic acid, in the hulls of several varieties of almonds.

#### 2.4.2. Identification of Volatile Compounds in *P. dulcis* Shell Extracts by GC-MS (before and after Derivatization)

The volatile profile of the different extracts was assessed using the GC-MS technique, as shown in the tables below (Table 3 and Table 4). The analysis by GC-MS method has led to the identification of 26 compounds before and after derivatization, among them seven compounds that had not been previously detected in the almond plant (2,3-dimethyldecane,undecane, 1,1′-bicyclohexyl, ben-zaldehyde, 3-hydroxy-4-methoxy, 2,4-Di-*tert*-butylphenol, dodecanoic acid, 1-methylethyl ester, and hydracrylic acid). Only 11 compounds were previously reported in almond shell part: dodecane, tridecane, hexanoic acid, glycolic acid, glyceric acid, butanedioic acid, malic acid, Dodecanoic acid, Pentadecanoic acid, 9-octadecenoic acid, (E)-, and stearic acid. As observed in Table 3 and Table 4, most of the identified molecules were found in the apolar extracts. Various classes of compounds are present in the almond shell extract, such as alkanes (undecane, dodecane, tridecane, etc.), organic acids (hexanoic acid, glycolic acid, butanedioic acid, etc.), and polyphenols (2,4-Di-tert-butylphenol). Some compounds, namely 2,4-Di-tert-butylphenol, benzaldehyde, 3-hydroxy-4-methoxy-, and palmitic acid, were found to be present in two or three of the extracts. According to the literature, only a limited number of studies have delved into the investigation of the volatile profile of the almond shell part. In this context, Moure et al. [9] identified several compounds in the almond shell part by the GC-MS method: fatty acids (butanedioic, octadecanoic, and trans-9 octadecenoic) and phenolic acids (vanillic, syringic, and p-coumaric acids). Furthermore, in another study several volatile compounds were identified in the *P. dulcis* shell, such as syringol, 1-(4-hydroxy-3-methoxyphenyl)-propyne, hexadecanoic acid, and syringaldehyde [6].

### 2.5. Biological Activities

#### 2.5.1. Antidiabetic Activity

The antidiabetic potential of the shell extracts was assessed in vitro using alpha-glucosidase and α-amylase assays.

**Figure 4:**
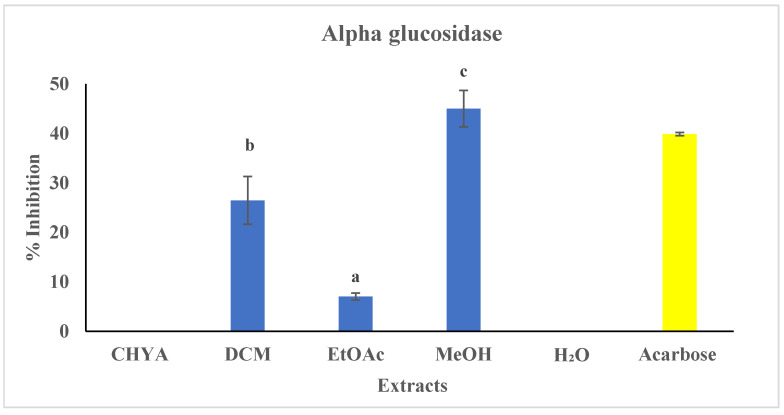
Antidiabetic activity (alpha-glucosidase) of different extracts of *P. dulcis* shell. Cyclohexane: CHYA; Dichloromethane: DCM; Ethyl acetate: EtOAc; Methanol: MeOH; Acarbose was used as a reference at 50 µg/mL. Extracts were tested at 50 µg/mL. Mean values ± SD (n = 3); Different letters on the histograms mean a significant difference (*p* < 0.05).

- α-glucosidase activity:

To the best of our knowledge, no previous studies have been conducted to assess the antidiabetic activity of the *P. dulcis* shell. The shell extracts were tested at 50 µg/mL The Acarbose was used in this assay as a reference at the same concentration. The results of the inhibition activity of the studied extracts are shown in Figure 4. As presented in the figure below, only three of the tested extracts have shown inhibitory activity against the alpha-glucosidase enzyme (DCM, EtOAc, and MeOH). The highest inhibition value was observed with the MeOH extract (45%), while the ethyl acetate extract presented the lowest one (7%). The observed variation in inhibitory activity among the extracts could potentially be correlated to their respective chemical compositions. Indeed, several bioactive compounds, especially the polar compounds, could be responsible for this activity, mainly the phenolic and polysaccharides. In the same context, a research conducted on the antidiabetic potential of almond kernel using the protein tyrosine phosphatase (1B PTP1B) inhibitory assay showed that seven alcoholic extracts exerted strong antidiabetic activity with an IC50 = 0.46 µg/mL with EtOH extract [1].

**Figure 5:**
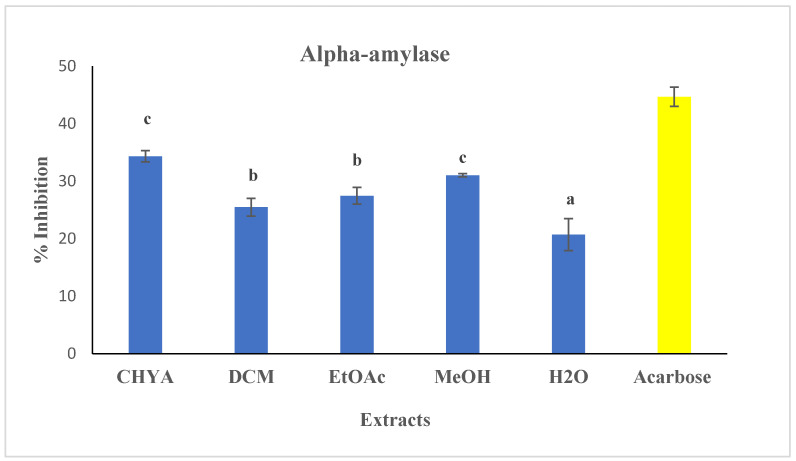
Antidiabetic activity (alpha-amylase) of different extracts of *P. dulcis* shell cyclohexane: CHYA; Dichloromethane: DCM; Ethyl acetate: EtOAc; Methanol: MeOH; Acarbose: was used as reference at 50 µg/mL). Extracts were tested at 50 μg/mL. Mean values ± SD (n = 3); Different letters on the histograms mean a significant difference (*p* < 0.05).

- Alpha-amylase assay:

The evaluation of the antidiabetic potential of almond shell extracts was performed using the alpha-amylase assay. The shell extracts and the reference concentration were tested at 50 µg/mL. The antidiabetic activity is illustrated in Figure 5. As shown in the extracts figure below, all the tested had an inhibition activity against the alpha-amylase enzyme with some variation. The highest inhibition value was recorded with the CHYA extract (34.34%), while the lowest value was recorded with the H_2_O extract (20.71%). In comparison with the alpha-glucosidase inhibition activity, we deduce that the polar shell extracts are more effective with the alpha-glucosidase assay while the apolar ones are more active with the alpha-amylase assay. These results could be explained by the difference in the inhibition mechanism between the two enzymes, as well as the compounds responsible for the inhibition activity. In the same context, Nowicka et al. [39] investigated the antidiabetic potential of several Prunus fruit smoothies and puree in vitro using the alpha glucosidase and the alpha amylase assays. The obtained results showed that the 100% apricot puree, 50% sour cherry juice and 50% apricot puree, and 20% sour cherry juice and 80% apricot puree exhibited the highest inhibition activity toward the alpha-amylase enzyme, with an IC_50_ < 1 mg/mL.

#### 2.5.2. Cytotoxic Activity

**Figure 6:**
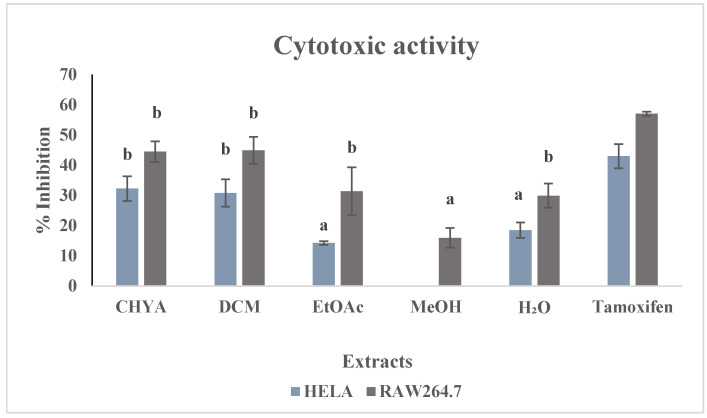
Cytotoxic activity of *P. dulcis* shell extracts against two cell types (HELA; RAW 264-7). Cyclohexane: CHYA; Dichloromethane: DCM; Ethyl acetate: EtOAc; Methanol: MeOH; Tamoxifen were used as references at 100 µM). Extracts were tested at 50 µg/mL Mean values ± SD (n = 3); Different letters on the histograms mean a significant difference (*p* < 0.05).

The cytotoxic potential of the obtained shell extracts was assessed by utilizing the MTT test method. Two cancer cell lines (HELA and Raw 264-7) were used in this study. The extracts were tested at 50 µg/mL the tamoxifen was used as a reference at 100 µM. The results of the inhibition activity were illustrated in Figure 6. As shown in the figure below, the different extracts exhibited moderate to low cytotoxic activity toward the two cell lines with a higher activity was demonstrated with the RAW 264-7. For the first cell line (HELA), we note that a significant difference was recorded between extracts with the highest inhibition activity with the apolar extracts (CHYA, DCM). Conversely, the MeOH extract did not exhibit any cytotoxic activity toward this cell line. For the second cell line (RAW 264-7), the apolar fractions presented the highest cytotoxic activity. These results suggest that apolar bioactive compounds could be responsible for this inhibition effect. Moreover, the shell extracts have demonstrated cytotoxic potential toward the two cell line. This could be explained by the presence of bioactive substances in the shell fractions capable of inhibiting the Hela and Raw 264-7 cells. In the same context, a study by Mericli et al. [40]), investigating the anticancer potential of *P. dulcis* seed oil against Colo-320 and Colo-741 cells, showed that the almond seed oil from northern Cyprus and Turkey varieties was an effective antiproliferative agent against the two cancer cell lines. In another study, Amico et al. [41] investigated the anticancer activity of terpenoids compounds obtained by bioguided fractionation assay of Sicilian almond hulls, and the results showed that betulinic acid, a compound isolated from almond hulls in this study, exhibited anticancer potential against the MCF-7 cell line with an antiproliferative activity GI50 = 0.27 μM.

### 2.6. Principal Component Analysis (PCA)

**Figure 7:**
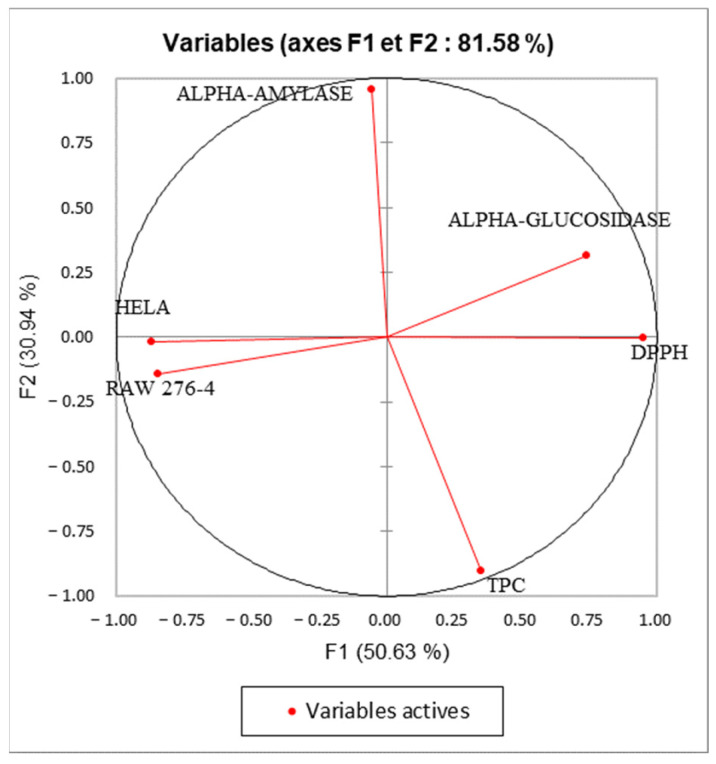
Principal components analysis (PCA) “loading plot” (TPC: total phenolic content), (DPPH: Antioxidant activity) and biological activities (Alpha-amylase, Alpha-glucosidase: Antidiabetic activity); (HELA and RAW 276-4: cytotoxic activity) of shell extracts of *P. dulcis*.

**Figure 8:**
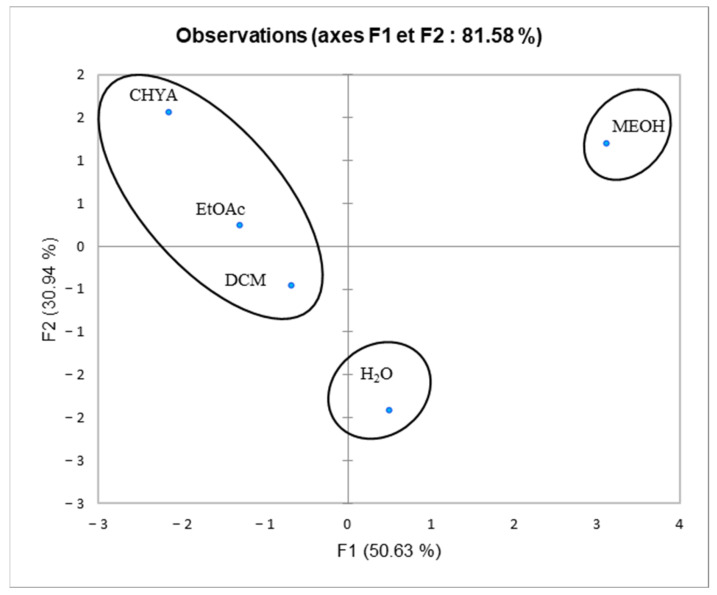
Principal component analysis (PCA) “score plot” of TPC: total phenolic content), (DPPH: antioxidant activity), and biological activities (Alpha-amylase, Alpha-glucosidase: Antidiabetic activity); (HELA and RAW 276-4: cytotoxic activity) of shell extracts of *P. dulcis*.

**Figure 9:**
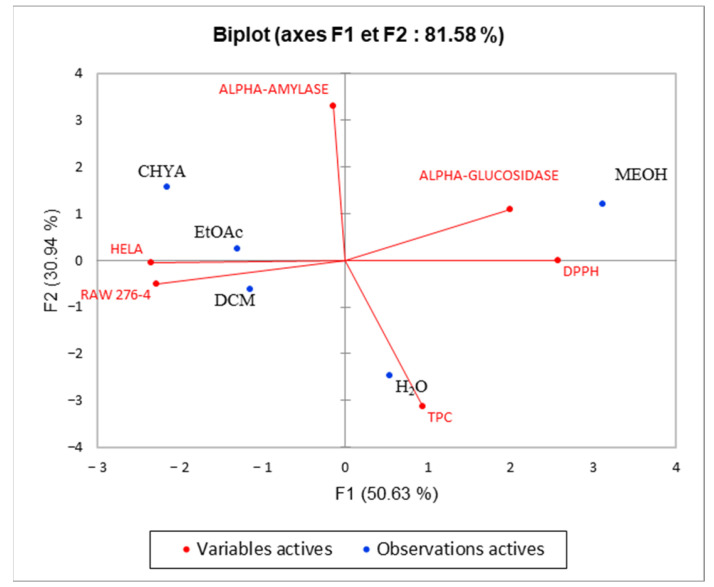
Principal components analysis (PCA) (“Biplot” of TPC: total phenolic content), (DPPH: antioxidant activity), and biological activities (Alpha-amylase, Alpha-glucosidase: Antidiabetic activity) (HELA and RAW 276-4: cytotoxic activity) of shell extracts of *P. dulcis*.

Principal component analysis was performed to determine the correlation between the TPC and the different biological assays. The inertia axes were withheld from this analysis. As shown in Figure 7, the total variation percentage recorded at 81.58%. The axes PC1 and PC2 expressed, respectively, 50.63% and 30.94% of variability. The “loading plot” (Figure 7) expressed the correlation between the tested activities, the TPC, and the correlation between the PC and the original variables. As presented in Table 5, PC1 is positively correlated with DPPH, alpha-glucosidase, Raw276-4, and HELA with loading 0.903, 0.547, 0.711, and 0.753, respectively. The PC2 is positively correlated with TPC and alpha-amylase with loading, respectively, 0.818, and 0.917. As shown in Figure 8, we can class the different extracts into three groups: C1 (MeOH), C2 (H_2_O), and C3 (CHYA, DCM, and EtOAc). Based on the correlation matrix (Table 6) and Figure 9, we note the presence of moderate correlation between the TPC and the DPPH (0.388) which suggests the presence of other compounds classes such as fatty acids or polysaccharides contributing to the antioxidant activity detected in the present study. Moreover, the TPC presents a negative correlation with (alpha-glucosidase, alpha-amylase, and cytotoxic activity against RAW 276-4 and HELA), which means that phenolic compounds present in the extracts could be not responsible for the obtained results and then, we can suggest the presence of other classes of compounds responsible for these effects such as the fatty acids, alkaloids, and polysaccharides. Furthermore, DPPH presents high correlation with alpha-glucosidase activity (0.680), suggesting that the substances exerting these effects could be the same. In addition, alpha-glucosidase presents a low correlation with alpha-amylase, which could indicate that these activities were exerted by different bioactive compounds, and let us suggest the presence of a different class of bioactive molecules in the shell extracts. For the cytotoxic activity, the correlation between the two-cell line is high (0.679). The correlation between observations and variables presented in Figure 9 shows that the MeOH extract is near to the DPPH and the alpha-glucosidase. This suggests that polar components such as polyphenols and polysaccharides may have a synergistic influence on the observed effects. In addition, the apolar fractions (CHYA, DCM, and EtOAc) are near to the alpha-amylase and the cytotoxic activities against RAW 276-4 and HELA, which suggests the presence of bioactive molecules in the mentioned fractions responsible for the present effects. In conclusion, based on the results of the biological assays and the statistical analysis, mainly the PCA, we can deduct that the shell extracts are rich in several bioactive compounds of different classes and polarity such as catechin, epicatechin, and synephrine, exerting moderate cytotoxic, antidiabetic, and antioxidant effects.

## 3. Materials and Methods

### 3.1. Plant Material

In this study, the shell material of *P. dulcis* (Achaak variety) was collected from the southeast of Tunisia (Zarzis). The collected plant material was air-dried then manually grounded (LGC France) and stored at room temperature.

### 3.2. Extraction

- Maceration:

Fifty grams of dried shell material was successively extracted, using organic solvents of increasing polarity cyclohexane (CHYA), dichloromethane (DCM), ethyl acetate (EtOAc), methanol (MeOH), and ultra-pure water (H_2_O) for 2 h with medium agitation at ambient temperature and pressure. The extraction mixture was subsequently filtered with Wattman paper. The solvents were then evaporated to dryness under a vacuum at 35 °C using a rotary evaporator (IKA, RV 10 auto V, Staufen, Germany).

### 3.3. Total Phenolic Content (TPC)

The total polyphenolic content of *P. dulcis* shell extracts was determined using the Folin–Ciocalteu method described by Ben Khadher et al. (2022) [42].

### 3.4. Determination of Radical Scavenging Activity (DPPH)

The DPPH assay was used in this study to assess the antioxidant scavenging activity of the different extracts as mentioned by Rahmani et al. [43], with some modifications. In the process, 20 µL of each diluted extract (0.5 mg/mL) was combined with 180 µL of a methanolic DPPH solution (0.2 N) in a 96-well microplate (Micro Well; Thermo Fisher Scientific, Illkirch, France). Following that, the reaction blend was subsequently incubated at 25 °C for a duration of 25 min. The absorbance was measured at 524 nm using a microplate reader (Multiskan Go F1-01620, Thermo Fisher Scientific, Vantaa, Finland). Ascorbic acid, at a concentration of 4 µg/mL, was used as the reference in this assay.

DPPH inhibition was calculated as follows: % Inhibition = 100 × (A_blank_ − A_sample_)/A_blank_.

### 3.5. Biological Activity

#### 3.5.1. Antidiabetic Activity

- Anti-α-Amylase Activity:

The anti-α-amylase activity of the shell extracts was performed as described by Ben khadher et al. [43].

- Anti-α-glucosidase Activity:

The anti-α-glucosidase activity of the different shell extracts was performed using the PNP-G method as described by Rahmani et al. [43]. In the reaction mixture, 50 µL of phosphate buffer (0.1 M, pH 6.9) was mixed with 100 µL of the alpha-glucosidase enzyme (1 U/mL) and 50 µL of each extract (0.5 mg/mL). After 10 min of incubation at 25 °C, 50 µL of PNP-G (5 mM) was added to the mixture. After 5 min of incubation, the absorbance was measured at 405 nm. The inhibition of enzyme activity was calculated as follows:

% inhibition = 100 × (A_blank_ − A_sample_)/A_blank_.

#### 3.5.2. Cytotoxic Activity

The cytotoxic activity of the shell extracts was determined on the cell line RAW 264.7 (mouse macrophage cell line) and Hela (human cervical cancer cell line), as described by Ben Khadher et al. [42] with slight modifications. For this process, cells were seeded into a 96-well microplate, with each well containing 13 × 10^3^ cells for Hela and 12 × 10^3^ cells for RAW 264.7 in a 100 µL volume. After 24 h of incubation at 37 °C, 100 µL of each extract diluted in the medium after being solubilized in DMSO was added to 100 µL of the corresponding culture medium; DMEM (Advanced DMEM, Thermo Fisher Scientific). The plate was then incubated for 48 h at 37 °C. The cytotoxic activity of the samples was evaluated using the MTT (3-(4,5-dimethylthiazol-2-yl)-2,5-diphenyltetrazolium bromide) assay. After removing the supernatant, cells were treated with 50 µL of MTT solution, then the plate was incubated at 37 °C for 40 min. MTT was then eliminated and 80 µL of DMSO was added. The absorbance was then recorded at 605 nm using the microplate reader (Mullikan Go, F1-01620, Thermo Fisher Scientific, Vantaa, Finland). The Tamoxifen at 100 µM was used in this assay as a reference.

### 3.6. Chromatographic Analysis

#### 3.6.1. High-Performance Liquid Chromatography Analysis (HPLC-DAD)

Analysis of the different shell extracts by HPLC-DAD was carried out in a Thermo Scientific Accela pump equipped with Accela PDA detector (280 nm wavelength was used for the detection of compounds). Separation was performed using an RP-C18 column (Phenomenex; Le Pecq, France). The dimensions of the column are 25 cm × 4.6 mm and particle size 5 µm. Elution was carried out with a flow rate of 0.5 mL/min. The mobile phase was made up of acidified water (pH = 2.65) as solvent A and acidified water/ACN (20:80 *v*/*v*) as solvent B. A linear gradient elution of the samples was followed: the solvent B concentration increased from 12% to 30% over 15 min, then escalated from 30% to 50% in 2 min, and finally from 50% to 99.9% in a span of 3 min. This was then dropped from 99.9% to 12% B within 7 min. Extracts were prepared at 10 mg/mL using acidified water/ACN (80:20 *v*/*v*) and filtered using a filter (Sigma Aldrich; Millex-HA filter 0.45 µm; Saint-Quentin-Fallavier, France). The identification and the quantification of the molecules was determined by comparison with the retention time and lambda max of known standards. Additionally, the quantity of each compound present in the shell extracts was determined using their specific calibration curves (Table 7).

The HPLC-DAD standards used in this analysis are: Trihydroxyethylrutin; rutin hydrate; catechin hydrate; 3,4-dihydroxy-5-methoxybenzoic acid; quercetin3-β-D-glucoside; polydatin; 2,4-dihydroxycinnamic acid; ellagic acid; synephrine; chlorogenic acid; green tea catechin; gallic acid; (−)epicatechin; gallocyanine; myricitrin dihydrate; brilliant yellow; methyl 3,5-dihydroxybenzoate; 3-amino-4-hydroxybenzoic acid; 3-amino-4-hydroxybenzoic acid; trans-3-hydroxycinnamic acid; *p*-coumac acid; sinapic acid; *trans*-ferulic acid; myricitrin; 4,7-dihydroxycoumarin; 7-hydroxycoumarin-3-carboxylic acid N-succinimidyl ester; 7-hydroxycoumarin-3-carboxylic acid; methyl 4-hydroxybenzoate; myricitrin; 6-hydroxycoumarin; coumarin; 7-hydroxy-4-methyl-3-coumarinylacetic acid; 3-cyanoumbelliferone; isopropyl 3,4,5-trihydroxybenzoate; resveratrol; 4-hydroxy-3-methoxycinnamic acid; 2-hydroxycinnamic acid; quercetin; ethyl 3,4-dihydroxycinnamate; 7,8-dihydroxy-2,2-dimethylchromane-6-carboxylic acid; *trans*-cinnamic acid;3.4-dihydroxy-benzoic acid methyl ester; 4-ethyl-7hydroxy-8-methyl-2H-chromen-2-one; α-cyano-4-hydroxycinnamic acid; naringenin; trolox; taxifolin hydrate; 7,3′-dihydroxyflavone; 2,4-dihydroxy-3,6-dimethylbenzoic acid; -6-hydroxy-2,5,7,8-tetramethylchroumane-2-carboxylic acid; butyl gallate; 6-hydroxyflavone; baicalein; ethyl 3,5-dihydroxybenzoate; ethyl trans-2-hydroxycinnamate; kaempferol; 5,8-dihydroxy-1,4-naphthoquinone; ethyl 4-hydroxy-3-cinamate; 7-hydroxy-4-phenylcoumarin; 2-chloro-3-(4-hydroxy-phenylamino)-(1,4)naphthoquinone; 3-chloro-7-hydroxy-4-methylcoumarin; 5-hydroxy-4′-methoxylflavone; chrysin; 4-hydroxy-3-(3-oxo-1-phenylbutyl)coumarin; icariin; 3′-hydroxy-a-naphthoflavone; 3-tert-butyl-4-hydroxybenzoic acid; 5,7-dihydroxy-3′,4′,5′-trimethoxyflavone; 7-hydroxyflavone; beta carotene; lutein; 4-hydroxytamoxifen; 5,7-dihydroxy-4-propylcoumarin; shikonin; 3′-hydroxy-6-methylflavone; 7-hydroxy-4-(trifluoromethyl)coumarin; 5-hydroxyflavone; isobutyl 4-hydroxybenzoate; 3,3′,4′_trimethoxyflavone; 4-hydroxy-3-propylbenzoic acid methyl ester; benzyl 4-hydroxybenzoate; 7-hydroxy-3′,4′,5′-trimethoxy-alpha-naphthoflavone; 3,3′-dimethoxyflavone; 2,3-dichloro-5,8-dihydroxy-1,4-naphthoquinone; 3,6,3′-trimethoxyflavone; 3,7-dimethoxyflavone; 5-hydroxy-3′-methoxyflavone; xanthurenic acid; 4′,5′-dimethoxy-2′-hydroxy-4-methylchalcone; (z)-3-(3-ethoxy-4-hydroxy-phenyl)-2-phenyl-acrylic acid; 2-chloro-3-(3,5-di-tert-butil-4-hydroxy-phenyl)(1-4)naphtoquinone; hamamelitannin; 9-chloro-10-hydroxy-2,3-dimethyl-anthracene-1,4-dione; 3,4-dihydroxy-5-methoxycinnamic acid; 5-hydroxy-7-((3-methylbenzyl)oxy)-2-phenyl-4h-chromen-4-one.

#### 3.6.2. Gas Chromatography-Mass Spectrometry (GC-MS) Analysis

The volatile composition of almond shell extracts was determined using the method mentioned by Rahmani et al. [43], with some modifications. The extracts obtained from shells were dissolved in their respective extraction solvents at a concentration of 3 mg/mL, with the exception of the water extract, for which methanol was used. The analysis was performed using a Saturn 2000 gas chromatograph (Les Ulis, France), which was outfitted with a fused silica capillary DB-5MS column (5% phenylmethylpolysiloxane, 30 × 0.25 mm, with a film thickness of 0.25 μm). H_2_ gas served as the carrier in this analytical procedure.

The chromatographic conditions were programmed as follows: an initial hold at 60 °C for 1 min, a subsequent increase to 150 °C at a rate of 10 °C/min, followed by a 1 min hold. A second gradient was then set to reach 260 °C at a rate of 12 °C/min, where it was held for 10 min. The temperature of the trap was maintained at 220 °C, while the transfer line was heated to 240 °C. Mass spectra were recorded within the range of 70 to 650 *m*/*z*. 5 µL of each prepared extract was injected.

- Derivatization method:

The derivatization method was conducted as described by Ben Khadher et al. [42].

- Compounds identification:

The volatile compounds present within the *P. dulcis* shell extracts have been identified through GC-MS, utilizing the Xcalibur software (version 3.0.63). The mass spectra and linear retention index (LRI) of each compound were then compared to those documented in the NIST MS library version 2.4, which was established on 25 March 2020.

### 3.7. Statistical Analysis

All experiments were conducted in triplicate. The data were expressed as means values ± standard deviations. The confidence limits were set at *p* < 0.05 and calculated according to the ANOVA test using the Statistical Package for the Social Sciences (SPSS) 22 (version IBM. 22.0. 2013; San Francisco, CA, USA). The difference between the extraction solvents was determined using the Tukey’s test. The principal component analysis (PCA) was conducted using XLSTAT (version 2021.3.1; Addinsoft; Pearson edition; Waltham, MA, USA).

## 4. Conclusions

The present study assesses the chemical profile and the biological potential of *P. dulcis* shell extracts. The chemical analysis led to the identification of 15 compounds by HPLC-DAD, of which 11 were firstly identified in the almond plant, and 26 volatile compounds by GC-MS. Among them, seven were identified for the first time in the *P. dulcis* plant. For the biological activities, shell extracts displayed moderate antioxidant, antidiabetic, and cytotoxic potential. Further studies are required to confirm the obtained results and to isolate the active compounds responsible for those activities.

## Figures and Tables

**Table 1:** Extraction yields of shell extracts of *P. dulcis* (DW%) (Cyclohexane: CHYA; Dichloromethane: DCM; Ethyl acetate: EtOAc; Methanol: MeOH).

	Fractional Extraction				
	CHYA	DCM	EtOAc	MeOH	H_2_O
**Maceration**	0.14	0.09	0.03	0.63	2.22

**Table 2:** Identification of compounds by HPLC-DAD of almond shell extracts. Cyclohexane: CHYA; Dichloromethane: DCM; Ethyl acetate: EtOAc; Methanol: MeOH.

Compounds	Rt (min)	Extracts					Ref.
		CHYA	DCM	EtOAc	MeOH	H_2_O	
Catechin	0.87				0.39		[10]
Rutin	0.93			13.06			[11]
Synephrine	1.09					1.62	[12]
Epicatechin	1.9				4.42		[13]
*trans*-3-hydroxycinnamic acid	3.5			0.08			[14]
*trans*-cinnamic acid	10.8					0.02	[15]
7,8-dihydroxy-2,2-dimethylchromane-6-carboxylic acid	10.93			0.31			[16]
5,7-dihydroxy-3′,4′,5′-trimethoxyflavone	19.51	0.31					[17]
3-tert-butyl-4-hydroxybenzoic acid	19.55				0.02		[16]
7-hydroxyflavone	19.71	0.59					[18]
Shikonin	20.72				0.19		[19]
Isobutyl 4-hydroxybenzoate	21.02	0.25					[16]
3,3′-dimethoxyflavone	21.59			0.23			[20]
3,6,3′-trimethoxyflavone	21.74			0.33		0.11	[21]
5-hydroxy-3′-methoxyflavone	21.95					0.33	[22]

**Table 3:** Identification of volatile compounds by GC-MS of almond shell extracts (before derivatization) Cyclohexane: CHYA; Dichloromethane: DCM; Ethyl acetate: EtOAc; Methanol: MeOH.

N	RI	Compounds	Area (10^7^)					Ref.
			Extracts					
			CHYA	DCM	EtOAc	MeOH	H_2_O	
1	1075	2,3-Dimethyldecane	12.6					[23]
2	1100	Undecane	0.0922					[24]
3	1205	Dodecane	6.76					[25]
4	1306	Tridecane	1.63					[26]
5	1377	1,1′-Bicyclohexyl	25,300	4.06				[26]
6	1535	Benzaldehyde, 3-hydroxy-4-methoxy-		370		4.99		[25]
7	1560	2,4-Di-tert-butylphenol	6.24	3.55	7.868			[27]
8	1566	Dodecanoic acid, 1-methylethyl ester		3.69				[28]

**Table 4:** Identification of volatile compounds by GC-MS of almond shell extracts (after derivatization) (CHYA: Cyclohexane; DCM: Dichloromethane; EtOAc: Ethyl acetate; MeOH: Methanol.

N	RI	Compounds	Area (10^7^)					Ref.
			Extracts					
			CHYA	DCM	EtOAc	MeOH	H_2_O	
1	1025	Cyclohexanol	15.4	4.02				[29]
2	1038	Furfuryl alcohol	3.13	14.3		39.1	23.4	[29]
3	1075	Lactic Acid		45.9		58.2	86.5	[30]
4	1094	Hexanoic acid		15.1				[30]
5	1094	Glycolic acid					121	[30]
6	1162	Hydracrylic acid			13.8	32.9		[31]
7	1266	Glycerol		88.9		1130	621	[26]
8	1338	Glyceric acid				41.7	109	[28]
9	1351	Butanedioic acid				97.9	0.691	[32]
10	1384	Nonanoic acid		51.6				[26]
11	1500	Malic acid				1760	1310	[33]
12	1591	2,4-Di-*tert*-butylphenol	22.2					[27]
13	1672	Dodecanoic acid		23.8				[34]
14	1865	Myristic acid	12.8					[34]
15	1959	Pentadecanoic acid	10.6					[33]
16	2054	Palmitic acid	511	4.60	2.84			[35]
17	2074	Myo-Inositol				428	1150	[36]
18	2241	9-octadecenoic acid, (E)-	278					[37]
19	2248	Stearic acid	165	121	392			[38]

**Table 5:** Correlations between variables and factors.

	F1	F2
TPC	0.121	0.818
DPPH	0.903	0.000
ALPHA-GLUCOSIDASE	0.547	0.100
ALPHA-AMYLASE	0.003	0.917
RAW 276-4	0.711	0.021
HELA	0.753	0.000

**Table 6:** Correlation matrix (Pearson (n)).

Variables	TPC	DPPH	ALPHA-GLUCOSIDASE	ALPHA-AMYLASE	RAW 276-4	HELA
TPC	1	0.388	−0.018	−0.828	−0.150	−0.209
DPPH	0.388	1	0.680	0.011	−0.757	−0.762
ALPHA-GLUCOSIDASE	−0.018	0.680	1	0.185	−0.490	−0.535
ALPHA-AMYLASE	−0.828	0.011	0.185	1	−0.099	0.063
RAW 276-4	−0.150	−0.757	−0.490	−0.099	1	0.679
HELA	−0.209	−0.762	−0.535	0.063	0.679	1

**Table 7:** Characterization of analytical standards detected in Prunus dulcis shell extracts: Retention times, calibration curves, and detection limits in HPLC analysis.

N°	Compound	RT	Calibration Curve	LOD (mg/L)	LOQ (mg/L)
1	Catechin	0.87	y = 0.7845x − 2.2956	0.055	0.181
2	Rutin	0.93	y = 0.1074x + 0.357	0.125	0.412
3	Synephrine	1.09	y = 0.385x + 0.5679	0.003	0.010
4	Epicatechin	1.9	y = 0.7845x − 2.2956	0.087	0.289
5	*trans*-3-hydroxycinnamic acid	3.5	y = 5.134x − 0.7396	0.002	0.008
6	*trans*-cinnamic acid	10.8	y = 5.134x − 0.7396	0.047	0.156
7	7,8-dihydroxy-2,2-dimethylchromane-6-carboxylic acid	10.93	y = 1.3215x + 2.8676	0.008	0.025
8	5,7-dihydroxy-3′,4′,5′-trimethoxyflavone	19.51	y = 0.2384x + 1.3435	0.087	0.289
9	3-tert-butyl-4-hydroxybenzoic acid	19.55	y = 0.4259x − 0.082	0.009	0.029
**10**	7-hydroxyflavone	19.71	y = 1.3215x + 2.8676	0.226	0.746
**11**	Shikonin	20.72	y = 0.1741x	0.011	0.037
**12**	Isobutyl 4-hydroxybenzoate	21.02	y = 0.4259x − 0.082	0.001	0.004
**13**	3,3′-dimethoxyflavone	21.59	y = 1.3215x + 2.8676	0.019	0.064
**14**	3,6,3′-trimethoxyflavone	21.74	y = 1.3215x + 2.8676	0.035	0.115
**15**	5-hydroxy-3′-methoxyflavone	21.95	y = 1.3215x + 2.8676	0.004	0.01

## Data Availability

Not applicable.
